# Dual-Mode Electrical–Optical Nanocomposite Hydrogel with Enhanced Upconversion Luminescence for Strain and pH Sensing

**DOI:** 10.3390/gels12040284

**Published:** 2026-03-28

**Authors:** Chubin He, Xiuru Xu

**Affiliations:** State Key Laboratory of Radio Frequency Heterogeneous Integration, College of Physics and Optoelectronic Engineering, Shenzhen University, Shenzhen 518060, China

**Keywords:** nanocomposite hydrogels, upconversion nanoparticles, dual-mode sensing, strain and pH sensors, flexible electronics

## Abstract

A dual-mode electrical–optical nanocomposite hydrogel is developed by integrating carboxyl-modified upconversion nanoparticles (UCNPs-COOH) and quaternized chitosan (CQAS) into a polyacrylamide (PAAm) covalent network. The hydrogel exhibits high optical transparency (>90% in the visible region), excellent mechanical properties (fracture strain of 1742%, tensile strength of 0.85 MPa, toughness of 6.57 MJ/m^3^), and robust adhesion to various substrates. The synergistic covalent–noncovalent hybrid network enables efficient energy dissipation, while CQAS-enhanced dispersion of UCNPs significantly improves upconversion luminescence intensity and stability, as evidenced by prolonged fluorescence lifetime from 0.564 ms to 0.691 ms at 539 nm. Leveraging distinct electrical and optical signal transduction pathways, the hydrogel functions as a highly sensitive resistive strain sensor with multistage gauge factors up to 13.85 and excellent cyclic stability over 1200 loading–unloading cycles at 100% strain for human motion monitoring. It also serves as a ratiometric optical pH sensor over a broad range (pH 1–13) based on phenolphthalein-sensitized upconversion luminescence, with excellent repeatability. By integrating real-time resistance responses with optical readouts within a single soft material, this work demonstrates a reliable dual-mode sensing strategy for simultaneous mechanical and chemical monitoring, holding promise for wearable electronics, smart healthcare, and environment-responsive sensing systems.

## 1. Introduction

The rapid advancement of flexible electronics has imposed increasing demands on next-generation intelligent sensing materials, particularly the need to emulate key characteristics of biological skin, such as mechanical compliance, adaptability, and the capability to simultaneously monitor diverse physical and chemical stimuli (e.g., strain, temperature, and pH) [1,2,3,4,5,6]. Among various candidate materials, conductive hydrogels have emerged as an attractive platform for flexible sensing applications owing to their three-dimensional polymer networks, excellent biocompatibility, tunable mechanical properties, and soft, hydrated nature that closely resembles biological tissues [7,8,9,10,11]. To date, conductive hydrogels based on carbon materials [12,13], metallic nanomaterials [14,15], or conductive polymers [16,17] have achieved considerable success in electrical signal transduction. However, these systems predominantly rely on a single electrical readout mode and typically exhibit limited optical functionality, while their electrical signals are often susceptible to environmental interference. These intrinsic limitations restrict their applicability in advanced multi-modal sensing scenarios.

In parallel, upconversion nanomaterials have attracted growing interest for integration into hydrogel systems due to their unique optical characteristics, including visible emission under near-infrared (NIR) excitation, high photostability, and deep tissue penetration [18,19,20,21]. Incorporation of upconversion nanoparticles (UCNPs) endows hydrogels with an additional optical signal transduction pathway, enabling optical monitoring of environmental changes—such as strain, temperature, or pH—through variations in emission intensity or wavelength [22,23,24]. For example, Huang et al. integrated NaYF_4_:Yb,Er nanoparticles exhibiting reversible temperature-dependent upconversion luminescence with poly(N-isopropylacrylamide) (PNIPAM) in a polyacrylamide hydrogel matrix to fabricate bifunctional hydrogels with high temperature sensitivity [25]. Xu et al. reported an injectable luminescent hydrogel composed of PLGA–PEG–PLGA triblock copolymers and NaYF_4_:Yb,Er hollow microtubules for noninvasive monitoring of bone regeneration [26]. Despite the encouraging progress in both conductive hydrogels and upconversion nanocomposites, existing systems often face inherent trade-offs among optical transparency, mechanical robustness, electrical conductivity, and multi-signal integration. Moreover, most reported hydrogel sensors rely on either electrical sensing or optical sensing. In particular, the challenge of achieving simultaneous real-time monitoring of mechanical and chemical signals, such as strain and pH, has yet to be adequately addressed in a soft, deformable, and biocompatible format.

This work presents a conceptually innovative design that integrates carboxyl-modified upconversion nanoparticles (UCNPs-COOH) and quaternized chitosan (CQAS) into a polyacrylamide (PAAm) matrix, forming a dual-mode electrical–optical nanocomposite hydrogel with synergistically enhanced functionality. Unlike previous systems that primarily rely on a single signal output, our hydrogel exploits two independent transduction pathways, resistive strain sensing and ratiometric optical pH sensing, enabling simultaneous monitoring of mechanical deformation and chemical environment changes within a single soft material platform. The incorporation of CQAS not only improves the dispersion and colloidal stability of UCNPs through electrostatic interactions but also contributes to ionic conductivity and interfacial adhesion, thereby mitigating aggregation-induced luminescence quenching in nanoparticle-doped hydrogels. Furthermore, the covalent–noncovalent hybrid network architecture imparts exceptional mechanical toughness (fracture strain of 1742%, toughness of 6.57 MJ/m^3^) and high optical transparency (>90%), which are challenging to achieve simultaneously in multifunctional hydrogel systems. This work provides a feasible strategy for integrating high-performance resistive strain sensing and phenolphthalein-sensitized upconversion luminescence-based ratiometric pH sensing within a single hydrogel platform, enabling detection across a wide pH range (pH 1–13). This dual-mode capability not only enhances sensing reliability by providing orthogonal signal readouts but also opens new possibilities for soft matter-based multimodal sensors in wearable healthcare, human–machine interfaces, and intelligent diagnostic systems.

## 2. Results and Discussion

### 2.1. Design and Preparation of PAAm/CQAS-UCNPs Nanocomposite Hydrogels

**Figure 1:**
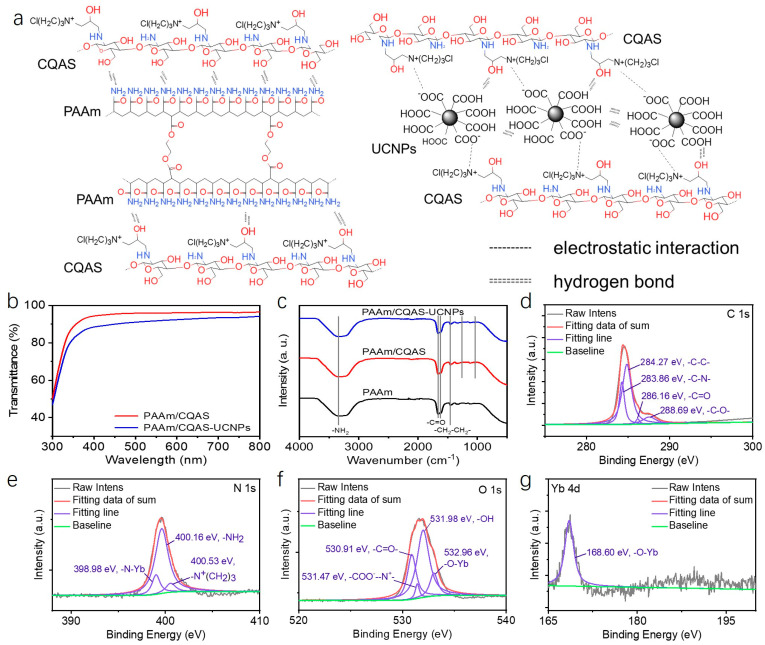
(**a**) Schematic illustration of the PAAm/CQAS-UCNPs nanocomposite hydrogel structure, featuring a covalent network formed by copolymerization of PAAm and poly(ethylene glycol) diacrylate (PEGDA), together with hydrogen bonding and electrostatic interactions among PAAm, CQAS, and UCNPs. (**b**) UV–Vis transmittance spectrum of the PAAm/CQAS-UCNPs nanocomposite hydrogel. (**c**) FT-IR spectroscopy of the PAAm/CQAS-UCNPs nanocomposite hydrogel. The high-resolution XPS spectra of PAAm/CQAS-UCNPs nanocomposite hydrogel for (**d**) C 1s, (**e**) N 1s, (**f**) O 1s and (**g**) Yb 4d.

Carboxyl-modified NaYF_4_:Yb,Er upconversion nanoparticles (UCNPs-COOH) were employed as the optical signal source, while quaternized chitosan (CQAS) was introduced as a dispersion stabilizer and ionic modifier. The UCNPs-COOH exhibited solid spherical or ellipsoidal morphologies with an average particle size of approximately 34 nm. Elemental analysis confirmed the presence of Na, Y, F, Yb, and Er within the nanoparticles (Figure S1). The UCNPs-COOH were dispersed separately in deionized water and in an aqueous CQAS solution, and their upconversion luminescence spectra were recorded (Figure S2). Characteristic upconversion emission peaks were observed at 527, 539, and 654 nm. Notably, UCNPs-COOH dispersed in the CQAS solution exhibited significantly enhanced luminescence intensity compared to those dispersed in pure water. This enhancement can be attributed to the combined effects of improved hydrophilicity resulting from carboxyl surface modification, which promotes homogeneous dispersion [27,28,29], and electrostatic interactions between positively charged CQAS chains and negatively charged UCNPs-COOH, which improve colloidal stability and suppress surface-related quenching effects [29,30]. Subsequently, acrylamide (AAm) monomers and poly(ethylene glycol) diacrylate (PEGDA) were introduced into the UCNPs-COOH dispersion, followed by a one-step thermally initiated free-radical polymerization to fabricate PAAm/CQAS-UCNPs nanocomposite hydrogels with upconversion luminescence. In this system, PAAm and PEGDA copolymerize to form a stable covalently crosslinked network, while hydrogen bonding, electrostatic interactions, and nanofiller effects among PAAm, CQAS, and UCNPs collectively generate a covalent–noncovalent hybrid network (Figure 1a).

This hybrid network provides effective energy dissipation during deformation, thereby contributing to the enhanced mechanical robustness of the nanocomposite hydrogel. The resulting hydrogels showed high optical transparency (Figure 1b), with transmittance exceeding 90% across the 400–800 nm wavelength range, which is favorable for optical signal readout. In addition, the stretching vibration peaks of the -C=O- group in the amide moiety appear at 1662 cm^−1^ and 1619 cm^−1^, the -NH_2_ stretching vibration of the amide group is observed at 3343 cm^−1^, and the -CH_2_-CH_2_- stretching vibration appears at 1448 cm^−1^. The peak at 1259 cm^−1^ is attributed to the stretching vibration of the β-1,4-glycosidic bond in CQAS, while the peak at 1049 cm^−1^ corresponds to the C-O-C stretching vibration of the glucopyranose ring in CQAS (Figure 1c). These results confirm the successful formation of the chemically crosslinked PAAm network and the effective incorporation of CQAS into the gel network. In addition, the presence of quaternary ammonium groups and mobile ions from CQAS, together with stable upconversion luminescence from UCNPs, enables the hydrogel to support both electrical and optical sensing functions. X-ray photoelectron spectroscopy (XPS) analysis was characterized, as shown in Figure 1d–g. The high-resolution C 1s spectrum (Figure 1d) shows characteristic peaks corresponding to C–C, C–N, C–O, and C=O bonds. In addition, the N 1s, O 1s, and Yb spectra (Figure 1e–g) confirm the coexistence of polymer functional groups and UCNP components within the hydrogel matrix. These results suggest the presence of electrostatic interactions between quaternary ammonium groups in CQAS and carboxyl groups on UCNPs-COOH, as well as coordination interactions between surface Yb ions and oxygen-containing groups in the polymer network. Such interfacial interactions are believed to contribute to the stabilization of UCNP dispersion and the mechanical robustness of the hydrogel. As schematically illustrated, the hydrogel can operate as a resistive strain sensor for human motion monitoring and as an upconversion resonance energy transfer-based optical pH sensor, forming a dual-mode electrical–optical sensing platform within a single soft material system.

### 2.2. Mechanical Properties, Ionic Conductivity, and Self-Adhesion of PAAm/CQAS-UCNPs Nanocomposite Hydrogels

**Figure 2:**
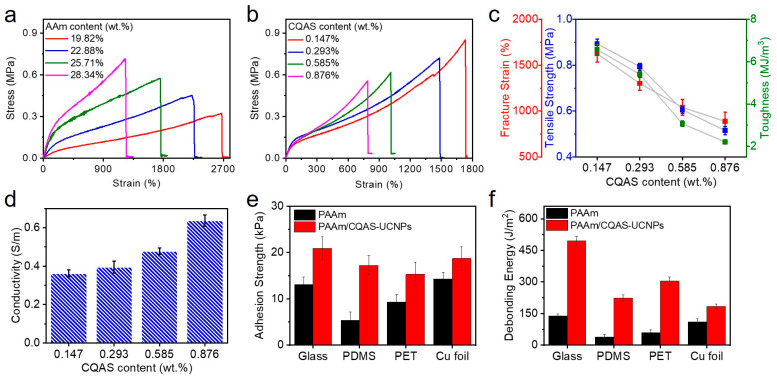
(**a**) Stress–strain curves of PAAm/CQAS-UCNPs nanocomposite hydrogels with different AAm contents. (**b**) Stress–strain curves of PAAm/CQAS-UCNPs nanocomposite hydrogels with different CQAS contents. (**c**) Fracture strain, tensile strength, and toughness of PAAm/CQAS-UCNPs nanocomposite hydrogels as a function of CQAS content. (**d**) Ionic conductivity of PAAm/CQAS-UCNPs nanocomposite hydrogels. (**e**) Adhesion strength of PAAm/CQAS-UCNPs nanocomposite hydrogels on various solid substrates. (**f**) Work of separation (peeling energy) of PAAm/CQAS-UCNPs nanocomposite hydrogels on different solid substrates.

Figure 2a presents the stress–strain curves of PAAm/CQAS-UCNPs nanocomposite hydrogels with different acrylamide (AAm) contents. As the AAm content increased from 19.82 wt.% to 28.34 wt.%, the tensile strength of the hydrogels increased from 0.34 MPa to 0.73 MPa, while the fracture strain decreased from 2674.8% to 1236.2%. The corresponding toughness initially increased and then decreased with increasing AAm content, reflecting the trade-off between network stiffness and extensibility. To further optimize the mechanical performance, CQAS was incorporated into the hydrogel matrix. Figure 2b shows the stress–strain curves of PAAm/CQAS-UCNPs nanocomposite hydrogels with varying CQAS contents, and the corresponding tensile strength, fracture strain, and toughness are summarized in Figure 2c. All three parameters exhibited maximum values at an optimized CQAS content, indicating that the introduction of CQAS effectively reinforces the hydrogel network. The Young’s modulus (E) was calculated from the initial linear region (0–10% strain) of the stress–strain curves in Figure 2b. As CQAS content increased from 0 to 0.5 wt.%, the modulus increased from 42 kPa to 52 kPa, representing a 23% enhancement. This increase correlates with the improved toughness (from 4.2 to 6.57 MJ/m^3^, a 56% increase), suggesting that CQAS contributes both to network stiffening through electrostatic interactions and to energy dissipation through reversible bond rupture. The enhancement in mechanical performance can be attributed to the formation of a covalent–noncovalent hybrid network, in which covalent crosslinks provide structural integrity, while reversible physical interactions contribute to effective energy dissipation during deformation. In addition, owing to the presence of polyionic groups and mobile ions from CQAS, the PAAm/CQAS-UCNPs nanocomposite hydrogels exhibit tunable ionic conductivity ranging from 0.36 to 0.63 S/m (Figure 2d), which is beneficial for stable electrical signal transduction under mechanical deformation.

Beyond mechanical robustness, the PAAm/CQAS-UCNPs nanocomposite hydrogels exhibit pronounced self-adhesive behavior. The adhesion–detachment characteristics of the hydrogels were evaluated using probe adhesion tests on various substrates, including glass, PDMS, PET, and copper foil. Further detailed adhesion behaviors are shown in Figure S3. Compared with pure PAAm hydrogels, the PAAm/CQAS-UCNPs nanocomposite hydrogels display larger deformation and higher detachment forces on all tested substrates, indicating enhanced interfacial adhesion. This improved adhesion performance arises from two primary factors. First, the single-network PAAm hydrogel lacks sufficient interfacial interactions with solid substrates and tends to undergo crack initiation and propagation during detachment, resulting in adhesive failure. In contrast, the PAAm/CQAS-UCNPs nanocomposite hydrogel benefits from an intrinsic energy dissipation mechanism associated with its hybrid network, which effectively dissipates interfacial fracture energy and suppresses crack propagation during peeling. Second, the abundant amino, hydroxyl, and cationic groups within the hydrogel matrix enable multiple physical interactions with substrate surfaces, including hydrogen bonding, electrostatic interactions, and metal coordination [31,32,33,34].

Based on the adhesion–detachment curves, the adhesion strength and work of separation (peeling energy) were quantified, as summarized in Figure 2e and Figure 2f, respectively. The adhesion strength was calculated as the peak detachment force divided by the contact area, while the peeling energy per unit area was determined from the integrated area under the force–displacement curve normalized by the contact area. The PAAm/CQAS-UCNPs nanocomposite hydrogels exhibit substantial adhesion strength and work of separation across multiple substrates, with adhesion strengths of 20.9, 17.2, 15.3, and 18.7 kPa and corresponding peeling energies of 495.3, 224.2, 305.2, and 184.6 J/m^2^ on glass, PDMS, PET, and copper foil, respectively. Such robust yet reversible adhesion is advantageous for maintaining reliable electrical and optical signal outputs in wearable and deformable sensing applications.

### 2.3. Upconversion Luminescence Properties of PAAm/CQAS-UCNPs Nanocomposite Hydrogels

**Figure 3:**
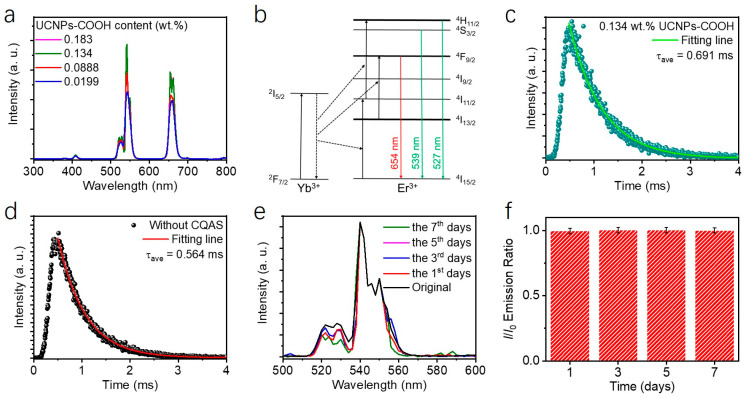
(**a**) Upconversion luminescence spectra of PAAm/CQAS-UCNPs nanocomposite hydrogels with different UCNPs-COOH contents under 980 nm excitation. (**b**) Schematic illustration of the upconversion process in the PAAm/CQAS-UCNPs nanocomposite hydrogel. (**c**) Fluorescence decay curve at 539 nm for PAAm/CQAS-UCNPs nanocomposite hydrogel containing 0.134 wt.% UCNPs-COOH under 980 nm excitation. (**d**) Fluorescence decay curve at 539 nm for PAAm/UCNPs hydrogel containing 0.134 wt.% UCNPs-COOH under 980 nm excitation. (**e**) Upconversion luminescence spectra of PAAm/CQAS-UCNPs nanocomposite hydrogel after storage at ambient temperature for 1, 3, 5, and 7 days. (**f**) Corresponding emission intensity at 539 nm as a function of storage time.

The upconversion luminescence properties of PAAm/CQAS-UCNPs nanocomposite hydrogels were investigated by varying the content of UCNPs-COOH, and the corresponding emission spectra are shown in Figure 3a. Under 980 nm laser excitation, the hydrogels exhibited bright green upconversion emission. As revealed in Figure 3a, characteristic emission peaks were observed at 527, 539, and 654 nm, which are assigned to the radiative transitions of Er^3+^ ions from the ^2^H_11/2_, ^4^S_3/2_ and ^4^F_9/2_ excited states to the ^4^I_15/2_ ground state, respectively [35], as schematically illustrated in Figure 3b. The upconversion emission spectra of the PAAm/CQAS–UCNPs hydrogel remained essentially unchanged when measured with and without electrical conduction (Figure S4), indicating that the electrical operation associated with strain sensing does not introduce detectable interference in the optical signal.

The emission intensity at 539 nm initially increased and then decreased with increasing UCNPs-COOH content (Figure 3a), exhibiting a non-monotonic dependence. This behavior can be understood in terms of two competing effects: improved emitter density at low nanoparticle loadings and concentration-induced quenching at higher loadings [36,37,38]. At relatively low UCNPs concentrations, increased nanoparticle incorporation reduces the relative contribution of surface defects and enhances upconversion efficiency. In contrast, beyond a critical UCNPs-COOH content of 0.134 wt.%, reduced interparticle spacing promotes surface-related quenching, leading to a decrease in emission intensity. Accordingly, the optimal luminescence intensity was achieved at a UCNPs-COOH content of 0.134 wt.%. To further elucidate the luminescence quenching behavior, fluorescence decay curves at 539 nm were measured under 980 nm excitation, and the corresponding average lifetimes were calculated. As shown in Figure 3c,d and Figure S5, the decay profiles could be well fitted using a bi-exponential function [39,40,41]:

(1) $$I\left(t\right)=I_{0}+A_{1}\exp\left(-t/\tau_{1}\right)+A_{2}\exp\left(-t/\tau_{2}\right)$$

where *I*(*t*) and *I*_0_ represent the emission intensity at time *t* and the initial intensity, respectively, A1 and A2 are fitting coefficients, and τ_1_ and τ_2_ correspond to the fast and slow decay components. The average fluorescence lifetime (τave) was calculated according to [40,41]:

(2) $$\tau_{ave}=\left(A_{1}\cdot \tau_{1}^{2}+A_{2}\cdot \tau_{2}^{2}\right)/\left(A_{1}\cdot \tau_{1}+A_{2}\cdot \tau_{2}\right)$$

As summarized in Figure 3c and Figure S5, the average fluorescence lifetimes at 539 nm for PAAm/CQAS-UCNPs nanocomposite hydrogels with different UCNPs-COOH contents were 0.684, 0.684, 0.691, and 0.661 ms, respectively. In comparison, the PAAm/UCNPs hydrogel without CQAS exhibited a markedly shorter lifetime of 0.564 ms (Figure 3d). This lifetime enhancement suggests that the incorporation of CQAS improves the dispersion and interfacial stability of UCNPs within the hydrogel matrix, thereby suppressing surface-related nonradiative quenching processes. The average fluorescence lifetime at 539 nm increased from 0.564 ms (without CQAS) to 0.691 ms (with 0.5 wt.% CQAS), corresponding to a 22.5% reduction in non-radiative decay rate (k_nr_ = 1/τ − k_r_ [42,43], assuming constant radiative rate k_r_). This suppression of non-radiative pathways is likely associated with reduced surface-related quenching resulting from improved colloidal stability. The energy transfer efficiency (η_ET_) between UCNPs and the surrounding matrix can be estimated using η_ET_ = 1 − τ_DA_/τ_D_, where τ_D_ is the lifetime in the absence of quenchers and τ_DA_ is the lifetime in the presence of quenchers. Comparing the PAAm/UCNPs hydrogel (τ = 0.564 ms, higher quenching) to the PAAm/CQAS-UCNPs hydrogel (τ = 0.691 ms, lower quenching), the relative reduction in quenching efficiency is approximately 18%. This suggests that CQAS effectively shields UCNPs from approximately one-fifth of the matrix-induced quenching effects. In addition, the luminescence stability of the PAAm/CQAS-UCNPs nanocomposite hydrogel was further evaluated by monitoring its emission behavior over a 7-day period under ambient conditions. As shown in Figure 3e,f, the emission intensity at 539 nm remained essentially unchanged during continuous monitoring, demonstrating excellent luminescence stability. This stability can be attributed to the presence of hydrophilic carboxyl groups on the UCNPs surface, which facilitate hydrogen bonding and electrostatic interactions with the PAAm/CQAS hybrid network, effectively preventing nanoparticle aggregation and ensuring sustained optical signal output.

Despite the significantly enhanced upconversion luminescence achieved in the PAAm/CQAS-UCNPs nanocomposite hydrogel, it is important to consider the potential adverse effects of the hydrogel matrix itself on the luminescence efficiency. The matrix is abundant in hydroxyl (-OH) groups, primarily originating from the aqueous medium and the polymer chains (e.g., from CQAS and the hydrated PAAm network). These -OH groups possess high-frequency vibrational modes (approximately 3300–3500 cm^−1^), which are energetically resonant with the energy gaps between the excited states of Er^3+^ ions (e.g., the ^4^S_3_/_2_ → ^4^I_15_/_2_ transition) [44]. This resonance facilitates efficient non-radiative relaxation of the lanthanide excited states through a multi-phonon relaxation process, where the electronic energy is dissipated as vibrational energy within the matrix [45,46]. This mechanism inherently competes with radiative emission and can substantially diminish the overall upconversion quantum yield. The experimental results indirectly support the mitigation of this effect through the strategic introduction of CQAS. The observed prolongation of the fluorescence lifetime at 539 nm—from 0.564 ms in the PAAm/UCNPs hydrogel to 0.691 ms in the PAAm/CQAS-UCNPs system—suggests a suppression of non-radiative pathways. This improvement can be attributed to the formation of an electrostatic complex between the positively charged CQAS chains and the negatively charged UCNPs-COOH. This interaction likely creates a physical barrier or a locally modified microenvironment around the nanoparticles, partially shielding them from direct contact with free water molecules and the abundant -OH groups of the polymer backbone. Consequently, the rate of -OH-induced surface quenching is reduced, leading to the observed enhancement in both luminescence intensity and lifetime.

In addition, to provide deeper insight into the luminescence enhancement mechanism, it is instructive to examine the behavior of individual Er^3+^ transitions. The green emission consists of two thermally coupled transitions: ^2^H_11_/_2_ → ^4^I_15_/_2_ (527 nm) and ^4^S_3_/_2_ → ^4^I_15_/_2_ (539 nm). These levels are separated by approximately 800 cm^−1^ and maintain a Boltzmann population distribution that is temperature-dependent but largely independent of surface effects [47,48]. The observation that both 527 nm and 539 nm emissions are enhanced upon CQAS incorporation (Figure S2) suggests that the enhancement is not transition-specific but rather reflects overall suppression of non-radiative relaxation pathways affecting all excited states.

However, the two green transitions exhibit different sensitivities to environmental perturbations under extreme pH conditions (Figure 5c). The stability of the 527 nm emission across pH 1–13 indicates that the ^2^H_11_/_2_ level is less susceptible to surface-related quenching, possibly due to its thermal coupling to ^4^S_3_/_2_ which maintains population equilibrium. In contrast, the 539 nm emission decreases under both acidic and alkaline extremes, suggesting that the ^4^S_3_/_2_ level is more sensitive to surface defects and ligand field changes. This differential sensitivity validates the use of 527 nm as an internal reference for ratiometric sensing, as it remains constant while 539 nm responds to environmental changes. The red emission at 654 nm (^4^F_9_/_2_ → ^4^I_15_/_2_) exhibits yet another behavior, increasing under alkaline conditions (Figure 5c). This increase cannot be explained by simple surface quenching and instead suggests pH-induced redistribution of population among Er^3+^ excited states. Possible mechanisms include (1) enhanced non-radiative relaxation from ^4^S_3_/_2_ to ^4^F_9_/_2_ due to altered phonon coupling; (2) changes in energy transfer pathways from Yb^3+^ to different Er^3+^ levels; or (3) inner filter effects from phenolphthalein preferentially attenuating green emission while leaving red emission unaffected.

Regarding the upconversion mechanism, NaYF_4_:Yb,Er is known to operate primarily through energy transfer upconversion (ETU), where Yb^3+^ ions absorb 980 nm photons and sequentially transfer energy to Er^3+^ ions [49,50]. Excited state absorption (ESA) plays a minor role due to the small absorption cross-section of Er^3+^ at 980 nm. The dominance of ETU is indirectly supported by the use of Yb^3+^ as a sensitizer at 20% doping concentration, which maximizes Yb^3+^-Er^3+^ interactions. The introduction of CQAS could affect ETU efficiency in several ways: (1) improved dispersion reduces interparticle energy transfer (cross-relaxation) that competes with ETU; (2) surface passivation reduces non-radiative relaxation from intermediate levels (^4^I_11_/_2_), increasing the probability of subsequent energy transfer steps; and (3) the charged polymer environment may alter the local crystal field, potentially affecting energy transfer rates.

### 2.4. PAAm/CQAS-UCNPs-Based Strain Sensors

**Figure 4:**
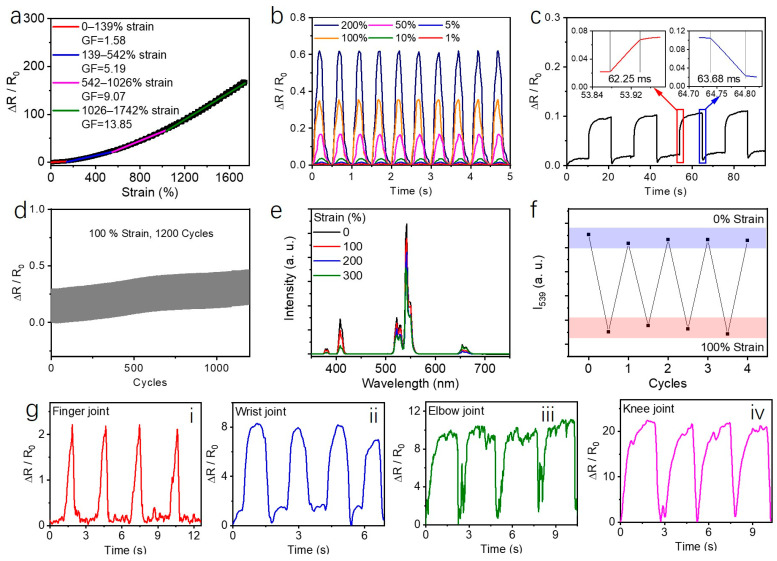
(**a**) Relationship between relative resistance change (*ΔR*/*R*_0_) and applied strain for the PAAm/CQAS-UCNPs nanocomposite hydrogel. (**b**) Relative resistance variation of the PAAm/CQAS-UCNPs nanocomposite hydrogel under different strain levels. (**c**) The response and recovery time of the PAAm/CQAS-UCNPs-based strain sensor. (**d**) Cyclic stability of the PAAm/CQAS-UCNPs hydrogel under repeated loading–unloading at 100% strain. (**e**) Upconversion luminescence spectra of the PAAm/CQAS-UCNPs hydrogel under different applied strains. (**f**) Luminescence intensity at 539 nm during four consecutive loading–unloading cycles at 100% strain. (**g**) Demonstration of the PAAm/CQAS-UCNPs nanocomposite hydrogel attached to different human joints for motion monitoring: finger (**i**), wrist (**ii**), elbow (**iii**), and knee (**iv**).

To take advantage of the excellent mechanical compliance and ionic conductivity of the PAAm/CQAS-UCNPs nanocomposite hydrogel, flexible strain sensors were fabricated, and the relationship between the relative resistance change (*ΔR*/*R*_0_) and applied tensile strain was systematically investigated, where R and *R*_0_ denote the resistance under strain and the initial resistance, respectively. The strain sensitivity was quantified using the gauge factor (GF), defined as the slope of the resistance change–strain curve [51,52]. As shown in Figure 4a, the relative resistance change increases continuously with increasing strain, indicating a stable and reproducible electrical response. Linear fitting analysis reveals that the strain–resistance curve can be divided into four characteristic regions, namely 0–139%, 139–542%, 542–1026%, and 1026–1742%, with corresponding gauge factors of 1.58, 5.19, 9.07, and 13.85, respectively. This multistage response behavior reflects the progressive evolution of conductive pathways within the hydrogel network under increasing deformation. Notably, the PAAm/CQAS-UCNPs strain sensor exhibits reliable signal outputs over both low-strain (1–10%) and high-strain (50–200%) regimes, demonstrating a broad and practical working strain range (Figure 4b).

The response and recovery behavior of the PAAm/CQAS-UCNPs-based strain sensor was characterized (Figure 4c). The sensor shows a response time of 62.25 ms and a recovery time of 63.68 ms, indicating its capability for transient strain detection. The performance of the sensor was further evaluated under different humidity conditions at 25 °C. The samples were kept at relative humidity levels of 50%, 75%, and 90% RH for 30 min before measurement to ensure stable conditions. Figure S6 shows that the sensor maintains stable and repeatable resistance signals over 10 loading–unloading cycles within a strain range of 0–100% at each humidity level, with no obvious signal drift. This suggests that the device can operate reliably under varying humidity environments. The cyclic stability of the sensor was then examined under repeated stretching. The relative resistance remains highly stable over more than 1200 cycles (Figure 4d), demonstrating good mechanical robustness and electrical reliability for long-term use in wearable sensing applications.

Importantly, the hydrogel also exhibits a coupled optical response under mechanical deformation. As shown in Figure 4e, the upconversion luminescence intensity at 539 nm gradually decreases with increasing applied strain. Moreover, the luminescence signal remains relatively stable during repeated loading–unloading cycles at 100% strain (Figure 4f), indicating that the hydrogel can provide simultaneous electrical and optical responses, which is beneficial for reliable dual-mode sensing. The cyclic loading–unloading tests at 100% strain (Figure 4d) demonstrate excellent electrical stability over 1200 cycles, with no significant baseline drift observed. This indicates that the ionic conductive pathways within the PAAm/CQAS–UCNPs hydrogel can reversibly reconstruct during repeated deformation. The hybrid network design plays a crucial role in this stability: the covalently crosslinked PAAm network provides elastic recovery, while reversible non-covalent interactions (hydrogen bonding and electrostatic interactions) enable efficient energy dissipation without permanent structural damage. Additionally, the ionic conductive pathways, facilitated by mobile ions from CQAS, can rapidly reform upon network relaxation due to the high mobility of ions in the hydrated environment. The stable upconversion luminescence under repeated loading–unloading cycles (Figure 4f) further confirms that the UCNPs remain homogeneously distributed without aggregation, supporting the overall structural integrity of the nanocomposite. To demonstrate practical applicability, the PAAm/CQAS-UCNPs-based strain sensor was attached to different human joints (Figure 4g), including the finger, wrist, elbow, and knee. Distinct and repeatable resistance signals were recorded during various joint movements, confirming the feasibility of the hydrogel sensor for real-time human motion monitoring.

### 2.5. PAAm/CQAS-UCNPs-Based pH Sensors

**Figure 5:**
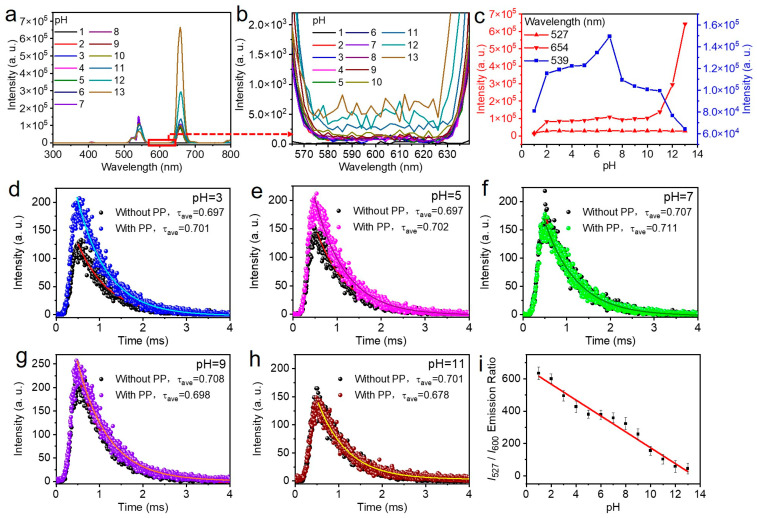
(**a**) Upconversion luminescence spectra of PAAm/CQAS-UCNPs nanocomposite hydrogels at different pH values. (**b**) Enlarged view of the corresponding spectra in the 560–640 nm wavelength range. (**c**) Emission intensities at 527 nm, 539 nm, and 654 nm as a function of pH. (**d**–**h**) Upconversion luminescence decay curves at 539 nm for PAAm/CQAS-UCNPs nanocomposite hydrogels with and without phenolphthalein at different pH values: (**d**) Ph = 3, (**e**) pH = 5, (**f**) pH = 7, (**g**) pH = 9, and (**h**) pH = 11. (**i**) Ratiometric pH response curve of the PAAm/CQAS-UCNPs hydro-gel-based sensor calculated as the luminescent emission ratio of *I*_527_/*I*_600_.

The PAAm/CQAS-UCNPs nanocomposite hydrogel was further integrated with phenolphthalein (PP) indicator to construct a self-referencing ratiometric pH sensor. Phenolphthalein is a well-known pH-responsive molecule that undergoes deprotonation under alkaline conditions, forming a red-colored quinoid structure with enhanced optical absorption [53]. Under 980 nm near-infrared excitation, partial upconversion emission from UCNPs at 539 nm can be transferred to phenolphthalein molecules through an upconversion resonance energy transfer (UC-RET) process in alkaline environments [54,55,56], leading to the appearance of a phenolphthalein-sensitized emission peak at approximately 600 nm (Figure 5a,b). In contrast, no sensitized emission at 600 nm is observed under neutral or acidic conditions, where phenolphthalein remains in its colorless lactone form (Figure 5b).

To elucidate the pH-dependent optical response, phenolphthalein was directly applied to PAAm/CQAS-UCNPs nanocomposite hydrogels equilibrated at different pH values, and the corresponding upconversion luminescence spectra were recorded. As shown in Figure 5a,c, the emission intensity at 539 nm exhibits pronounced pH dependence. Specifically, the intensity decreases with decreasing pH under acidic conditions, while it also diminishes with increasing pH in alkaline environments. The observed decrease in 539 nm emission under extreme pH conditions may arise from a combination of factors: surface-related quenching of UCNPs, pH-induced network structural changes affecting UCNP local environment and light extraction, and inner filter effects under alkaline conditions. In comparison, the emission at 654 nm remains relatively stable under neutral and acidic conditions (pH ≤ 7) but increases progressively under alkaline conditions (pH > 7) (Figure 5c), accompanied by the emergence and growth of the sensitized emission peak at 600 nm (Figure 5b). These results indicate that phenolphthalein in its deprotonated quinoid form reabsorbs part of the upconverted emission, attenuating the 539 nm signal while generating a new pH-dependent optical output [54].

In addition, the pH-dependent changes in relative emission intensities (Figure 5c) provide additional insight into the energy level dynamics of Er^3+^. Under alkaline conditions (pH > 7), the 539 nm emission decreases while the 654 nm emission increases, resulting in a significant increase in the red-to-green ratio (*I*_654_/*I*_539_). This behavior suggests that the population distribution among Er^3+^ excited states is altered, not merely the overall quantum yield. Several mechanisms could account for this redistribution. First, pH-induced changes in the surface chemistry of UCNPs-COOH may alter the local crystal field symmetry, affecting the relative probabilities of radiative transitions from different levels. Second, the presence of deprotonated phenolphthalein may introduce wavelength-dependent inner filter effects or preferential energy transfer to specific Er^3+^ levels. The stability of the 527 nm emission argues against simple wavelength-independent attenuation, supporting more complex state-specific effects.

The upconversion luminescence decay curves at 539 nm were measured for PAAm/CQAS-UCNPs hydrogels with and without phenolphthalein (PP) at different pH values. As shown in Figure 5d–h, all decay curves can be well fitted using a bi-exponential model Equation (1), and the corresponding average fluorescence lifetimes (τ_ave_) were calculated using Equation (2). At pH ≤ 7, the average lifetimes of hydrogels with and without phenolphthalein are nearly identical (Figure 5d–f), indicating negligible energy transfer under neutral or acidic conditions. This behavior is attributed to the non-conjugated lactone structure of phenolphthalein, which limits its ability to accept energy from the upconversion emission. In contrast, under alkaline conditions (pH > 7), phenolphthalein-containing hydrogels exhibit consistently shortened fluorescence lifetimes compared to those without phenolphthalein. For example, at pH= 9 and 11, the average lifetimes decrease from 0.708 ms and 0.701 ms (without PP) to 0.698 ms and 0.678 ms (with PP), respectively (Figure 5g,h). This lifetime reduction provides evidence for the occurrence of UC-RET from the nanocomposite hydrogel to phenolphthalein in alkaline environments, enabled by the extended π-conjugated quinoid structure.

To discuss the pH sensing mechanism, several additional factors warrant consideration. Variations in local ion concentration (H^+^, Na^+^, Cl^−^) accompany the adjustment of pH values and may influence the optical properties of the hydrogel matrix. However, such effects are expected to be minimal because the hydrogel remains highly transparent across the investigated pH range. More importantly, the sensing mechanism is closely associated with the structural transition of phenolphthalein. Under alkaline conditions (pH > 7), phenolphthalein undergoes deprotonation to form a quinoid structure with an extended π-conjugated system, which exhibits strong absorption around ~550 nm. This spectral overlap enables two parallel processes. First, upconversion resonance energy transfer (UC-RET) can occur from excited UCNPs to nearby phenolphthalein molecules, resulting in sensitized emission near 600 nm and a reduction in the fluorescence lifetime at 539 nm. Second, the strong absorption of the quinoid form may introduce an inner filter effect, which further attenuates the green emission through radiative reabsorption. The fluorescence lifetime data (Figure 5d–h) support a dynamic quenching mechanism under alkaline conditions, as evidenced by reduced lifetimes. Nevertheless, the possibility of additional contributions, such as static quenching or pH-dependent adsorption of phenolphthalein on the UCNP surface, cannot be completely excluded. Taken together, a comprehensive mechanism can be proposed in which the quinoid form of phenolphthalein enables both UC-RET and inner filter effects under alkaline conditions, leading to the observed ratiometric optical response.

Notably, the emission intensity at 527 nm remains nearly constant across the entire pH range investigated (Figure 5c), allowing it to serve as an internal reference signal. Accordingly, a ratiometric pH sensing strategy was established based on the intensity ratio between the upconversion emission at 527 nm and the phenolphthalein-sensitized emission at 600 nm (*I*_527_/*I*_600_). As shown in Figure 5i, the hydrogel-based pH sensor exhibits a linear response over a broad pH range from 1 to 13. The sensitivity (S) was calculated as the slope of the linear region of the *I*_527_/*I*_600_ vs. pH curve (Figure 5i): S = Δ(*I*_527_/*I*_600_)/ΔpH = 0.42 per pH unit over the range pH 1–13.

To verify the reproducibility of the ratiometric response, 20 independent PAAm/CQAS-UCNPs-based pH sensors were tested at pH = 9. As shown in Figure S7, the emission spectra and the corresponding ratiometric intensity values (*I*_527_/*I*_600_, *I*_539_/*I*_600_, and *I*_654_/*I*_600_) remain highly consistent with negligible variation, indicating good reproducibility of the sensor response. Similar reproducibility was also observed at pH = 4 (Figure S8), further confirming the reliability of the sensing strategy under different pH conditions. In addition, the energy transfer efficiency (η_RET_) under alkaline conditions was calculated from the fluorescence lifetime data using η_RET_ = 1 − τ_PP_/τ_noPP_, where τ_PP_ and τ_noPP_ are the lifetimes with and without phenolphthalein. At pH = 9, η_RET_ = 1 − 0.698/0.708 = 1.4%; at pH = 11, η_RET_ = 1 − 0.678/0.701 = 3.3%. The increase in η_RET_ with pH correlates with the increasing concentration of deprotonated quinoid phenolphthalein, which has a larger spectral overlap integral with UCNP emission. Using Förster resonance energy transfer (FRET) theory, the average distance (r) between UCNPs and phenolphthalein molecules can be estimated from r = R_0_(1/η_RET_ − 1)^1^/^6^, where R_0_ is the Förster distance (typically 3–6 nm for UCNP-dye pairs). Assuming R_0_ ≈ 4 nm, the calculated distances are r ≈ 8.2 nm at pH = 9 and r ≈ 6.7 nm at pH = 11, consistent with surface-localized energy transfer. To highlight the sensitivity of our hydrogel in strain sensing performance, we compared it with other advanced hydrogel strain sensors [57,58,59,60,61,62]. As shown in Table 1, our gel exhibits excellent light transmittance and mechanical properties, while also demonstrating superior dual-modal sensing performance, with a comparable gauge factor relative to several previously reported hydrogel strain sensors.

It is important to clarify the working mode of phenolphthalein in this pH sensing system. As described in Section 4.7, the phenolphthalein indicator is not incorporated into the hydrogel network during synthesis but is instead externally applied to the pre-formed hydrogel surface via drop-casting, followed by 5 min of absorption staining. This configuration represents a single-use, surface-adsorption working mode, analogous to pH test paper, where the indicator is temporarily deposited onto the test medium rather than permanently embedded within it. Consequently, systematic optimization of phenolphthalein concentration within the hydrogel matrix was not performed in this study, as the indicator is not intended to reside within the bulk network. The working mechanism relies on the pH-dependent structural transition of surface-adsorbed phenolphthalein (lactone ↔ quinoid form) rather than concentration-dependent effects within the matrix. This approach offers distinct advantages, including simplicity, rapid sample screening (5 min staining), and versatility for use with different indicators, while reducing potential concerns about indicator leaching during storage. Compared to literature systems employing covalently embedded indicators, which enable reversible and reusable pH sensing with precisely controlled loading, our single-use configuration prioritizes ease of operation and rapid multi-sample screening over reusability. The 20 samples measured at pH = 9 showing relatively constant peak values (Figures S7 and S9) confirm excellent measurement repeatability under this working mode. These results demonstrate that the PAAm/CQAS-UCNPs nanocomposite hydrogel enables reliable optical pH sensing while maintaining its favorable mechanical and electrical properties, highlighting its potential for multifunctional sensing applications.

## 3. Conclusions

In summary, a multifunctional PAAm/CQAS–UCNPs nanocomposite hydrogel was developed that integrates high optical transparency, mechanical robustness, stable ionic conductivity, and dual electrical–optical sensing capabilities within a single soft material platform. The introduction of quaternized chitosan effectively improves the dispersion and interfacial stabilization of UCNPs, resulting in enhanced upconversion luminescence intensity and long-term optical stability. Meanwhile, the synergistic combination of a covalently crosslinked PAAm network with multiple non-covalent interactions gives rise to a hybrid network structure that supports efficient energy dissipation, mechanical resilience, and robust adhesion to diverse substrates.

Benefiting from these integrated structural and functional features, the hydrogel enables reliable resistive strain sensing for human motion monitoring as well as ratiometric optical pH sensing over a broad range from pH 1 to 13 based on phenolphthalein-sensitized upconversion luminescence. This work demonstrates a practical strategy for constructing dual-mode soft sensors by coupling mechanical compliance with optical functionality in hydrogel systems, offering new opportunities for the design of multifunctional sensing materials. Despite these promising attributes, several limitations and challenges must be addressed to facilitate the translation of this hydrogel platform from laboratory prototypes to real-world applications. First, the long-term operational stability of the hydrogel under dynamic and humid environments remains to be systematically evaluated. While the material exhibits excellent luminescence stability over seven days under ambient conditions, its performance under continuous mechanical cycling, variable temperatures, or prolonged exposure to extreme pH conditions warrants further investigation. Second, the current fabrication process relies on batch-style thermal polymerization, which may hinder scalability and reproducibility for industrial production. Developing continuous manufacturing techniques, such as UV-initiated polymerization or microfluidic-assisted synthesis, could enhance throughput and consistency. Moreover, the integration of dual-signal readouts—electrical and optical—into a single device poses challenges in terms of signal decoupling, data synchronization, and miniaturization of interrogation systems. For wearable applications, the development of flexible, lightweight, and wireless readout modules compatible with both resistive and luminescent sensing modalities is essential. Additionally, the biocompatibility and potential cytotoxicity of UCNPs and quaternized chitosan components must be thoroughly assessed through in vitro and in vivo studies, particularly for implantable or long-term wearable applications. Looking forward, the design principles established in this work provide useful guidance for the development of advanced soft materials with integrated electrical and optical sensing functionalities. Future efforts should focus on enhancing environmental robustness, establishing scalable fabrication routes, and engineering integrated device architectures that support real-time, multiplexed sensing in complex service environments. With continued interdisciplinary collaboration, such multifunctional hydrogel systems hold great potential for transformative applications in wearable electronics, smart healthcare, human–machine interfaces, and beyond.

## 4. Materials and Methods

### 4.1. Preparation of PAAm/CQAS-UCNPs Nanocomposite Hydrogels

Carboxyl-modified NaYF_4_:Yb,Er upconversion nanoparticles (UCNPs-COOH) aqueous dispersion (10 mg mL^−1^) was purchased from Nanjing XFNANO Materials Tech Co., Ltd. (Nanjing, China). Acrylamide (AAm, A.R.), poly(ethylene glycol) diacrylate (PEGDA, average M_n_ ≈ 400), ammonium persulfate (APS, A.R.), hydrochloric acid (HCl, A.R.), and sodium hydroxide (NaOH, A.R.) were obtained from Shanghai Aladdin Biochemical Technology Co., Ltd. (Shanghai, China). Quaternized chitosan (CQAS, substitution degree ≥ 98%, deacetylation degree ≥ 90%, purity ≥ 95%, molecular weight 50–100 kDa) was supplied by Anhui Kuer Bio-engineering Co., Ltd. (Anhui, China).

A precursor solution was prepared by dissolving AAm (1.50 g), CQAS (0.025 g), and PEGDA (0.50 μL) in deionized water (5.00 g) under continuous stirring at room temperature for 2 h. Subsequently, predetermined amounts of UCNPs-COOH were added to the solution and dispersed uniformly by ultrasonication for 30 min. After addition of APS (0.03 g) as the thermal initiator and thorough mixing, the precursor solution was poured into molds and thermally polymerized at 80 °C for 30 min to obtain PAAm/CQAS-UCNPs nanocomposite hydrogels.

### 4.2. Physical Characterizations

The morphology and size of NaYF_4_ upconversion nanoparticles were characterized using field-emission transmission electron microscopy (FETEM; Tecnai G2 F30, FEI, Hillsboro, OR, USA). Elemental composition was analyzed by energy-dispersive X-ray spectroscopy (EDS) equipped on the TEM system. The chemical structures of PAAm/CQAS-UCNPs nanocomposite hydrogels were examined by Fourier transform infrared spectroscopy (FT-IR; Spectrum One Version B, PerkinElmer, Waltham, MA, USA). Optical transmittance of the hydrogel samples was measured using a UV–Vis–NIR spectrophotometer (UV-3600PLUS, Shimadzu, Tokyo, Japan).

### 4.3. Mechanical Properties of PAAm/CQAS-UCNPs Nanocomposite Hydrogels

Hydrogel specimens were cut into rectangular strips with dimensions of 21 mm × 5 mm × 0.4 mm. Uniaxial tensile tests were performed at room temperature using a universal testing machine (CTM2010, Xieqiang Instruments, Shanghai, China). All measurements were conducted at a constant crosshead speed of 100 mm min^−1^, and at least five specimens were tested for each formulation. Stress–strain curves were recorded to determine mechanical parameters. The elastic modulus was calculated from the slope of the initial linear region of the stress–strain curve, while the toughness was obtained by integrating the area under the stress–strain curve.

### 4.4. Self-Adhesive Properties of PAAm/CQAS-UCNPs Nanocomposite Hydrogels

Self-adhesive performance of the hydrogels was evaluated using a probe adhesion test. Hydrogel samples (1 cm × 1 cm × 1 cm) were fixed onto the surface of a testing probe with cyanoacrylate adhesive (502 glue) and mounted on a universal testing machine (CTM2010, Xieqiang Instruments, Shanghai, China). The hydrogel was brought into contact with various substrates, including glass, PDMS, PET, and copper foil, at an approach speed of 10 μm s^−1^ until a preload force of 1 N was reached. After maintaining contact for 5 s, the probe was retracted at the same speed. The adhesion–detachment process was recorded as force–displacement curves. Adhesion strength and work of separation (peeling energy) were calculated from the peak detachment force and the integrated area under the force–displacement curve, respectively.

### 4.5. Ionic Conductivity and Strain-Sensing Performance

Electrical resistance of the hydrogel samples was measured using a digital multimeter (DMM6500, 6½-Digit, Keithley, Solon, OH, USA). Rectangular hydrogel strips (21 mm × 5 mm × 0.4 mm) were prepared, and copper wires were attached to both ends of each sample to serve as electrodes. The electrical conductivity (*σ*) was calculated according to

σ = 1/*ρ* = *L*/(*R* × *S*) (3)

where *L* is the sample length, *R* is the measured resistance, and *S* is the cross-sectional area. Strain-sensing performance was evaluated by synchronizing the universal testing machine (CTM2010, Xieqiang Instruments, Shanghai, China) with the digital multimeter. During tensile deformation, resistance changes and applied strain were recorded simultaneously, enabling real-time acquisition of resistance–strain response curves.

### 4.6. Upconversion Luminescence Characterization

Upconversion luminescence properties of the PAAm/CQAS-UCNPs nanocomposite hydrogels with different UCNPs-COOH contents and pH values were characterized using a steady-state/transient fluorescence spectrometer (FLS1000, Edinburgh Instruments, Livingston, UK). For strain-dependent optical measurements, hydrogel samples were mounted on a miniature tensile stage and stretched to predetermined strain levels prior to spectral acquisition.

### 4.7. PAAm/CQAS-UCNPs-Based pH Sensors

For the preparation of hydrogels at different pH values, the pH of the precursor solution was adjusted to the desired values using dilute HCl or NaOH solutions before the polymerization and curing step, while all other preparation conditions remained unchanged. For pH-responsive luminescence evaluation, two drops of phenolphthalein reagent were added to the hydrogel surface. After 5 min of absorption and staining, the upconversion luminescence spectra were collected.

## Figures and Tables

**Table 1:** Comparison of key properties of representative hydrogel sensors reported in the literature.

Materials	Optical Transmittance (%)	Fracture Strain (%)	Tensile Strength (MPa)	Toughness (MJ/m^3^)	GF	pH Sensing Performance	Refs.
PDA-rGO, CMC-Na, PAAm, Fe(NO_3_)_3_	Opaque	1225	-	0.826	6.44	pH-responsive swelling	[57]
P(AAm-co-MA), Fe(NO_3_)_3_	-	400–500	1.2	3.32	3	pH-triggered shape-memory behavior	[58]
P(AA-co-MAA), ODex, FeCl_3_	-	556	0.063	-	5.5	pH-triggered shape-memory behavior	[59]
rGO@PDA, HGG, Fe^3+^, Glycerin, Borax	-	728	0.42	-	11.3	-	[62]
PAAm, PAA, F-MOF, PS MPC	-	-	-	-	-	Linear fluorescence–pH correlation over the pH range of 3–7	[60]
Agarose, DHLA-Ag NCs	Transparent	-	-	-	-	pH-dependent fluorescence response over the pH range of 4–8	[61]
PAAm/CQAS-UCNPs	Transparent (>90%)	1733.33	0.85	6.57	13.85	ratiometric pH sensing over a broad pH range (1–13)	This work

Note: PDA-rGO, polydopamine-reduced graphene oxide, CMC-Na, carboxymethyl cellulose sodium; PAAm, polyacrylamide, P(AAm-co-MA), poly(acrylamide-co-maleic acid); P(AA-co-MAA), poly(acrylic acid-co-2-methylallylamine), ODex, oxidized dextran; rGO, reduced graphene oxide; Fe^3+^, ferric chloride; PDA, polydopamine; HGG, hydroxypropyl guar gum; PAA: poly(acrylic acid); F-MOF: fluorescent metal–organic framework (CDs@UiO-66(OH)_2_); PS MPC, polystyrene microsphere photonic crystal; DHLA-Ag NCs, dihydrolipoic acid-capped silver nanoclusters. GF values correspond to the maximum gauge factor reported for each sensor.

## Data Availability

The raw data supporting the conclusions of this article will be made available by the authors on request.

## Supplementary Materials

The following supporting information can be downloaded at: https://www.mdpi.com/article/10.3390/gels12040284/s1, Figure S1. (a) High-resolution TEM image and (b) Elemental analysis of carboxyl-modified NaYF_4_:Yb,Er upconversion nanoparticles (UCNPs-COOH). Figure S2. Upconversion luminescence spectra of UCNPs-COOH in pure water and in CQAS aqueous solution. The UCNPs-COOH dispersed in the CQAS aqueous solution demonstrated significantly enhanced luminescence intensity compared to those in pure water. Figure S3. Displacement-time and force-time curves for the adhesion behavior tests of PAAm hydrogel and PAAm/CQAS-UCNPs nanocomposite hydrogel on different solid substrates: (a) glass, (b) PDMS, (c) PET, and (d) copper foil. Compared to PAAm hydrogel, the PAAm/CQAS-UCNPs nanocomposite hydrogel exhibits greater deformation and stronger adhesion forces across various substrates. Furthermore, comparative experimental results reveal that the PAAm/CQAS-UCNPs hydrogel demonstrates higher peeling forces and greater deformation on rigid substrates such as glass and copper foil than on flexible substrates including PDMS and PET. Figure S4. Upconversion emission spectra of the PAAm/CQAS–UCNPs nanocomposite hydrogel measured under electrical conduction (closed-circuit condition) and without electrical conduction. Figure S5. Fluorescence decay curves at 539 nm under 980 nm excitation for PAAm/CQAS-UCNPs nanocomposite hydrogels with different UCNPs-COOH contents: (a) 0.0199 wt.% UCNPs-COOH, (b) 0.0888 wt.% UCNPs-COOH, (c) 0.183 wt.% UCNPs-COOH. The corresponding fluorescence lifetime is 0.684 ms, 0.684 ms, and 0.661 ms, respectively. Figure S6. Relative resistance variation of the PAAm/CQAS-UCNPs-based strain sensor at different humidity levels (50%, 75%, and 90% RH) at 25 °C over 10 loading–unloading cycles within a strain range of 0–100%. Figure S7. Reproducibility of the ratiometric response of the PAAm/CQAS-UCNPs-based pH sensor evaluated using 20 independent samples at pH = 9. (a) Upconversion emission spectra of the sensor. (b) Corresponding ratiometric intensity values (*I*_527_/*I*_600_, *I*_539_/*I*_600_, and *I*_654_/*I*_600_) as a function of sample number (1–20). Figure S8. Reproducibility of the ratiometric response of the PAAm/CQAS-UCNPs-based pH sensor evaluated using 20 independent samples at pH = 4. (a) Upconversion emission spectra of the sensor. (b) Corresponding ratiometric intensity values (*I*_527_/*I*_600_, *I*_539_/*I*_600_, and *I*_654_/*I*_600_) as a function of sample number (1–20). Figure S9. Reproducibility of the PAAm/CQAS-UCNPs-based pH sensor evaluated using 20 independent samples at pH = 9. The emission intensity at 539 nm extracted from the spectra in Figure S7a is plotted as a function of sample number (1–20).
