# Supplementary material for: Dual-Mode Electrical–Optical Nanocomposite Hydrogel with Enhanced Upconversion Luminescence for Strain and pH Sensing
Source: Gels. 2026 Mar 28;12(4):284. doi: 10.3390/gels12040284 (PMC13115103; doi:10.3390/gels12040284)
Supplement: Supplementary file 1 [file gels-12-00284-s001.zip › gels-4177194-supplementary.pdf]

Supporting Information

Dual-Mode Electrical–Optical Nanocomposite Hydrogel with Enhanced Upconversion Luminescence for Strain and pH Sensing

Chubin He and Xiuru Xu *

State Key Laboratory of Radio Frequency Heterogeneous Integration, College of Physics and Optoelectronic Engineering, Shenzhen University, Shenzhen 518060, China

* Correspondence: xiuruxu@szu.edu.cn

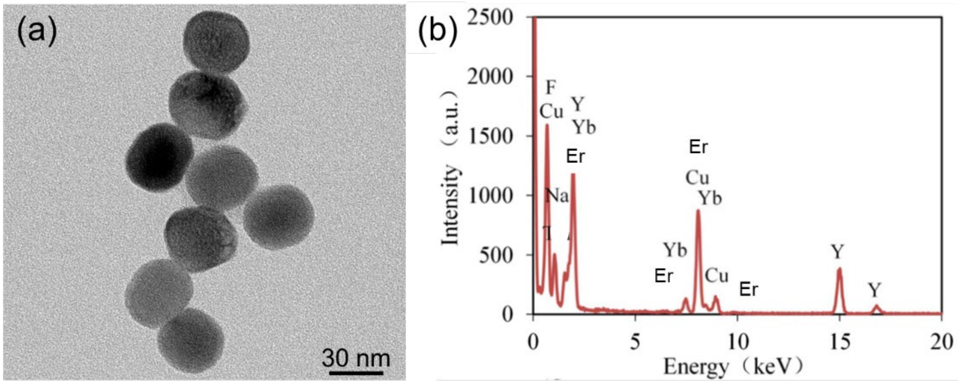

Figure S1. (a) High-resolution TEM image and (b) Elemental analysis of carboxyl-modified $NaYF_4$:Yb,Er upconversion nanoparticles (UCNPs-COOH).

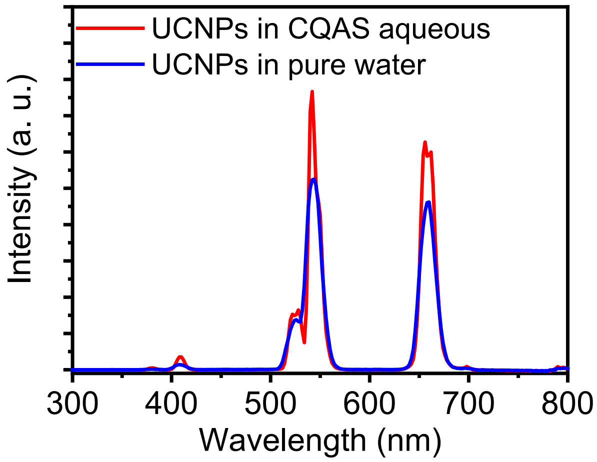

Figure S2. Upconversion luminescence spectra of UCNPs-COOH in pure water and in CQAS aqueous solution. The UCNPs-COOH dispersed in the CQAS aqueous solution demonstrated significantly enhanced luminescence intensity compared to those in pure water.

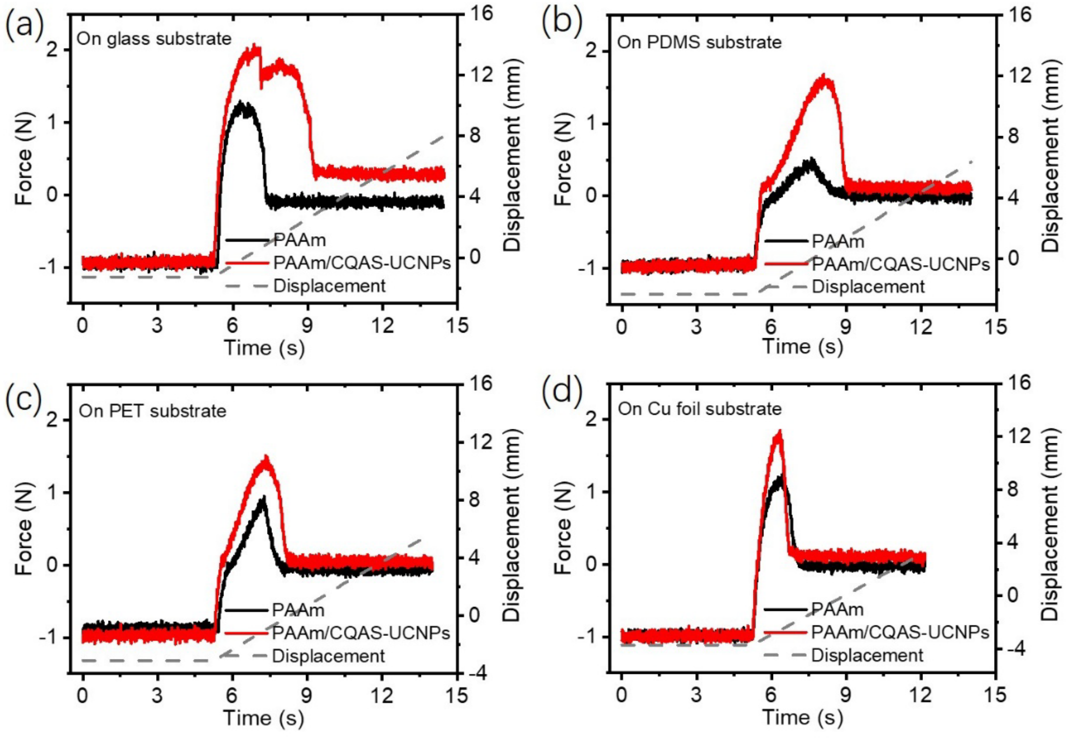

Figure S3. Displacement-time and force-time curves for the adhesion behavior tests of PAAm hydrogel and PAAm/CQAS-UCNPs nanocomposite hydrogel on different solid substrates: (a) glass, (b) PDMS, (c) PET, and (d) copper foil. Compared to PAAm hydrogel, the PAAm/CQAS-UCNPs nanocomposite hydrogel exhibits greater deformation and stronger adhesion forces across various substrates. Furthermore, comparative experimental results reveal that the PAAm/CQAS-UCNPs hydrogel demonstrates higher peeling forces and greater deformation on rigid substrates such as glass and copper foil than on flexible substrates including PDMS and PET.

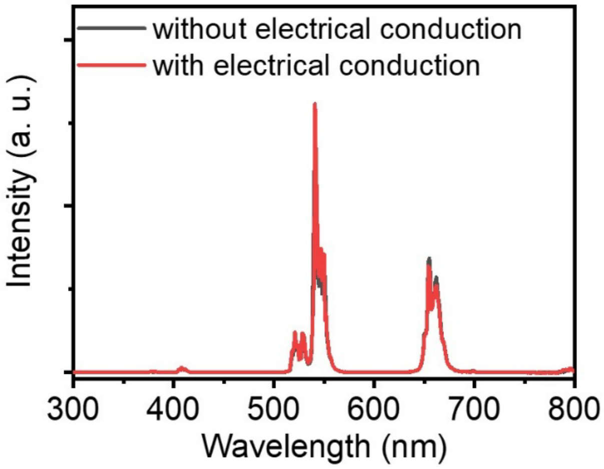

Figure S4. Upconversion emission spectra of the PAAm/CQAS–UCNPs nanocomposite hydrogel measured under electrical conduction (closed-circuit condition) and without electrical conduction.

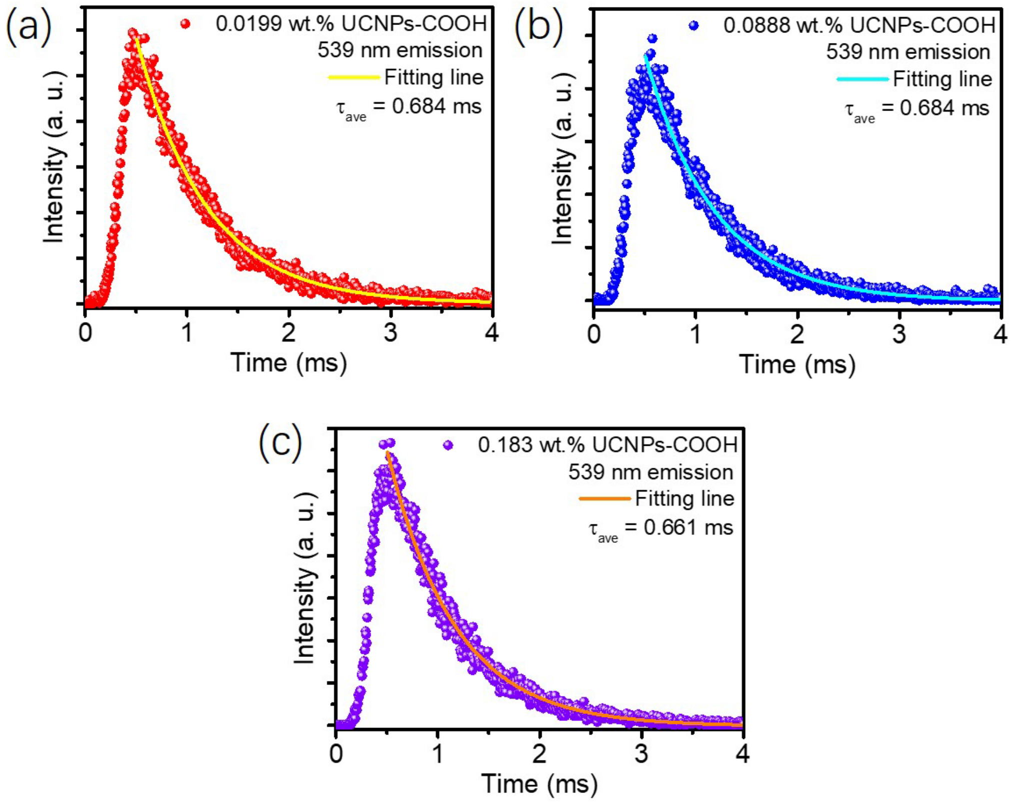

Figure S5. Fluorescence decay curves at 539 nm under 980 nm excitation for PAAm/CQAS-UCNPs nanocomposite hydrogels with different UCNPs-COOH contents: (a) 0.0199 wt.% UCNPs-COOH, (b) 0.0888 wt.% UCNPs-COOH, (c) 0.183 wt.% UCNPs-COOH. The corresponding fluorescence lifetime is 0.684 ms, 0.684 ms, and 0.661 ms, respectively.

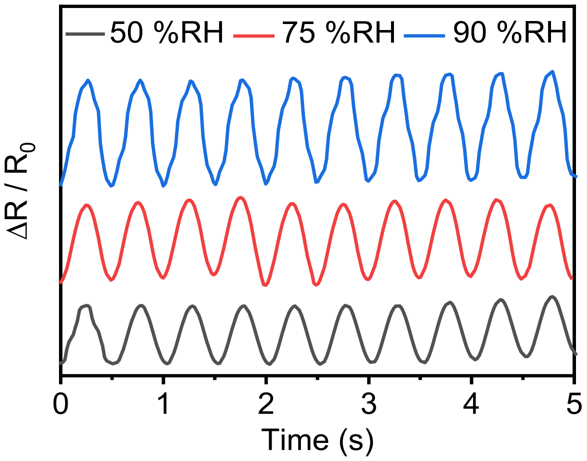

Figure S6. Relative resistance variation of the PAAm/CQAS-UCNPs-based strain sensor at different humidity levels (50%, 75%, and 90% RH) at 25 °C over 10 loading-unloading cycles within a strain range of 0–100%. The results demonstrate stable and repeatable electrical responses under varying humidity conditions.

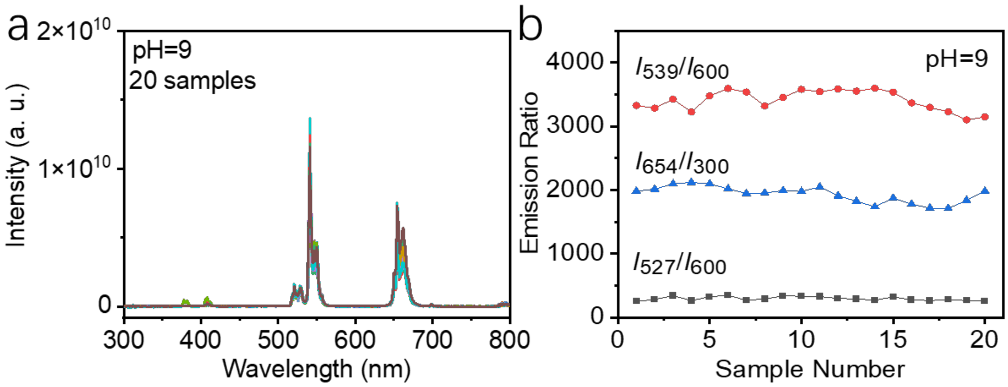

Figure S7. Reproducibility of the ratiometric response of the PAAm/CQAS-UCNPs-based pH sensor evaluated using 20 independent samples at pH = 9. (a) Upconversion emission spectra of the sensor. (b) Corresponding ratiometric intensity values (I_{527}/I_{600}, I_{539}/I_{600}, and I_{654}/I_{600}) as a function of sample number (1–20).

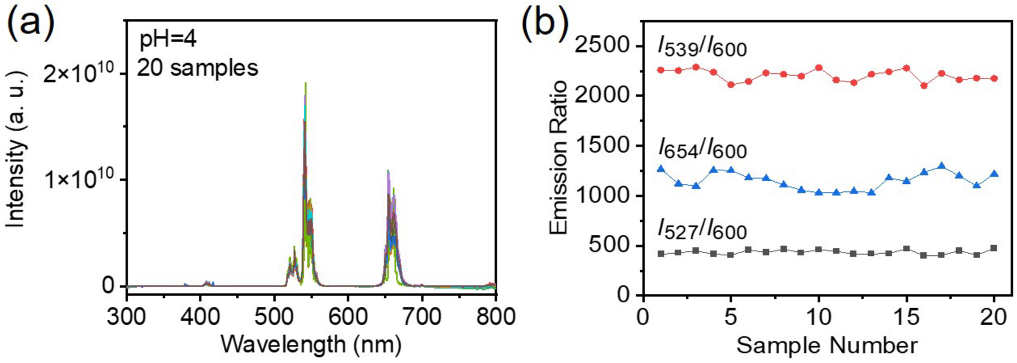

Figure S8. Reproducibility of the ratiometric response of the PAAm/CQAS-UCNPs-based pH sensor evaluated using 20 independent samples at pH = 4. (a) Upconversion emission spectra of the sensor. (b) Corresponding ratiometric intensity values (I_{527}/I_{600}, I_{539}/I_{600}, and I_{654}/I_{600}) as a function of sample number (1–20).

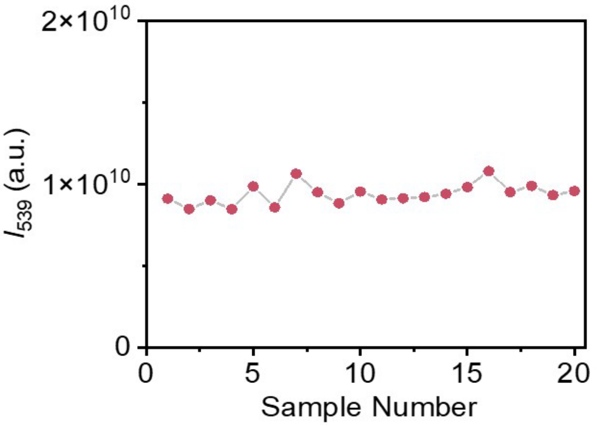

Figure S9. Reproducibility of the PAAm/CQAS-UCNPs-based pH sensor evaluated using 20 independent samples at pH = 9. The emission intensity at 539 nm extracted from the spectra in Figure S7a is plotted as a function of sample number (1–20).
